# Serum Fibroblast Growth Factor 21 in Patients with and without Pterygia

**DOI:** 10.18502/jovr.v15i1.5940

**Published:** 2020-02-02

**Authors:** Gholamhosein Yaghoobi, Saeed Shokoohi-Rad, Hamid Jafarzadeh, Elham Abdollahi

**Affiliations:** ^1^Ophthalmology Department, Birjand University of Medical Sciences, Valiasr Hospital, Birjand, Iran; ^2^Eye Research Center, Mashhad University of Medical Sciences, Mashhad, Iran; ^3^Birjand University of Medical Sciences, Birjand, Iran

**Keywords:** Fibroblast, Growth Factor 21, Pterygium, Serum Levels

## Abstract

**Purpose:**

Pterygium is a common fibro-vascular-related eye disease. The fibroblast growth factor 21 (FGF21) helps reduce neovascularization. Previous studies have shown that the serum level of FGF21 correlates with vascular eye diseases such as diabetic retinopathy and retinopathy of prematurity. In this study, the serum FGF21 is compared in patients with and without pterygium.

**Methods:**

This descriptive-analytical cross-sectional study examines individuals with pterygium who visited the Ophthalmology Clinic of Khatam-al-Anbia Hospital in Mashhad, Iran, during 2017–2018. Control subjects were selected from healthy people without pterygium disease. Patients with a history of acute illness, chronic liver and kidney disease, diabetes, cancer, malnutrition and drug use, women who were pregnant or breastfeeding, and subjects who were taking anticonvulsants or glucocorticoids were excluded as these may affect insulin and glycosuria levels. Sixty people (30 in each group) were chosen using the convenient sampling method. Intravenous blood samples were taken from all patients. After preparing the patients, the freeze was checked using the enzyme-linked immunosorbent assay (ELISA) method after samples had been taken. Data were analyzed by SPSS using an independent *t*-test, Mann–Whitney, Chi-square, Kruskal–Wallis, and Kolmogorov–Smirnov tests (α = 0.05).

**Results:**

The serum FGF21 levels were 319.09 ± 246.93 pg/ml and 608.88 ± 449.81 pg/ml (*P* = 0.005) in the pterygium group and control subjects, respectively. The average serum FGF21 was 281.55 ± 40.74 pg/ml in males and 361.375 ± 10.298 pg/ml in females in the pterygium group. The difference was not statistically significant (*P* = 0.19)

**Conclusion:**

Our study showed that FGF21 levels were lower in patients with pterygium than the control subjects to a statistically significant level.

##  INTRODUCTION

Pterygium thickens on bulbar conjunctiva as a prominent triangular mass and extends to the cornea. This condition involves degeneration of the elastoid collagen and invades the cornea with subepithelial fibrovascular tissue. This disease occurs in specific geographical zones with varying prevalence; with outdoor work increasing its incidence by 1.5 times. The reason for this is unknown. Generally, pterygium is considered a trivial problem unless it adversely affects the visual axis, which is dependent on its location. However, it can be concerning for patients due to its unusual and inflammatory appearance.^[1,2]^ The decision to undergo surgery varies according to the growth speed of the mass and the degree of astigmatism. It is recommended to avoid surgery for only the cosmetic reasons.^[3]^ Despite numerous methods for treating pterygium, there is a 30–70% recurrence rate, which drives ocular surgeons to use adjunct medicines.^[3]^

**Table 1:** Comparison of mean age and gender in pterygium and control groups


**Group**	**Pterygium patients**	**Control patients**	**Independent ** ***t*** **-test**
Variable	Mean ± SD	Mean ± SD	*t* = 1.8
Age	42.21 ± 12.32	38.28 ± 10.83	*p* = 0.08
Gender			*X${}^{2}$* = 0.27
Male	17(56.7)	15(50.0)	*p* = 0.6
Female	13(43.3)	15(50.0)	
Total	30(100.0)	30(100.0)	
SD, standard deviation			

**Table 2:** Comparison of mean serum FGF21 in patients with pterygium in terms of gender


**Group**	**Male**	**Female**	**Mann–Whitney test**
Variable	Mean ± SD	Mean ± SD	
Serum FGF21	281.55 ± 40.74	361.375 ± 10.298	Z = 2.95
			p = 0.198


**Table 3:** Comparison of mean cholesterol, creatinine, urea, SGOT, SGPT, HDL, and LDL between the pterygium and control groups


**Group**	**Pterygium patients**	**Control patients**	**Independent ** ***t*** **–test / Mann–Whitney test**
**Variable**	**Mean ± SD**	**Mean ± SD**	
**Cholesterol **	206.86 ± 50.77	155.53 ± 32.15	t = 4.67
		*p* < 0.0001
**Triglyceride **	138.66 ± 102.83	98.90 ± 50.52	Z = 2.17
		*p* = 0.03
**LDL**	101.71 ± 30.62	76.00 ± 22.26	*t* = 3.60
		*p* = 0.001
**HDL**	48.17 ± 22.03	57.33 ± 33.7	Z = 1.28
		*p* = 0.2
**Creatinine**	0.92 ± 0.18	0.95 ± 0.14	Z = 1.28
		*p* = 0.2
**Urea**	28.07 ± 7.94	24.43 ± 6.86	*t* = 1.58
		*p* = 0.07
**SGOT**	26.58 ± 9.96	21.96 ± 6.90	Z = 1.67
		*p* = 0.09
**SGPT**	28.15 ± 17.14	23.21 ± 9.81	Z = 0.77
		*p* = 0.42
HDL, high-density lipoprotein; LDL, low-density lipoproteins; SD, standard deviation; SGOT, serum glutamic-oxaloacetic transaminase			

The fibroblast growth factor (FGF) group includes 22 members, each of which is divided into seven subfamilies based on homology and phylogeny. The subgroups of FGF19 including FGF15, FGF21, and FGF23 play a significant role in endocrine performance and metabolizing carbohydrates and lipids.^[4]^ Growth factors play an important role in many ocular diseases. In a study on mice, the administration of FGF21, which is a member of this family, reduced angiogenesis through adiponectin. In doing so, FGF21 increases adiponectin and as a result, decreases TNFα.^[5,6]^ The serum level of FGF21 is reduced in diabetes type I but is increased in diabetes type II. The unusual increase of FGF21 in diabetes type II is a compensatory protective response^[7]^ that induces hypoglycemia and improves sugar metabolism in the body.^[8,9,10]^ Recent studies have indicated that serum FGF21 is lower in patients with type I diabetes mellitus and in neonates with retinopathy of prematurity compared to a healthy control group.^[8,10,11]^ Moreover, some studies indicated that the administration of FGF21 reduced LDL, triglycerides, and cholesterol and increased HDL.^[9]^ Hence, FGF21 has a significant impact on improving obesity- and diabetes-related complications.^[8]^ Considering the prevalence of pterygium, the fibrovascular nature of pterygium, the protective effects of FGF21 on angiogenesis, and the lack of published studies so far, the present researchers decided to compare the serum FGF21 in the blood samples of patients with and without pterygium.

##  METHODS

The study population included patients with clinical signs of pterygium who had presented to the Ophthalmology Clinic of Khatam-Al Anbia Hospital in Mashhad, Iran, during 2017–2018. All volunteered to participate in the study, were not under any drug regimen, and had no specific disease. The control group included patients without pterygium who presented to the aforementioned clinic and accepted to participate in the study. They also were not under any drug regimen, had no underlying disease, and were matched for age and gender with the case group [Table 1]. Patients who did not wish to participate or had a history of acute illness, chronic liver and kidney disease, diabetes, cancer, malnutrition, drug use, were pregnant, breastfeeding, or were taking anticonvulsants or glucocorticoids were excluded as these may affect insulin and glycosuria levels. Participants were selected using the convenient sampling method. Patients diagnosed with pterygium by an ophthalmologist were assigned to the case group. The same number of subjects who matched the case group in terms of age and gender were assigned to the control group. Given the lack of any similar study, we used a sample volume of 30 patients per group as a pilot study. Having selected the case and control subjects, 5 cc of venous blood was taken by a phlebotomist from the radial vein of each participant after fasting for 12 h. The blood samples were first centrifuged for 20 min at 3000 rpm, and the overlying serum was isolated. Care was taken to have samples that were not severely hemolyzed, blurred, lipemic, or infected with microbes. The isolated sera were divided into small portions and kept at –20${}^{\circ }$C until the testing began. The samples were examined using the enzyme-linked immunosorbent assay (ELISA) method with special kits for human studies (China Crystaldi Co., purchased from Pardisa Tebazema Co., Mashhad, Iran). Then, the total serum rate of FGF21 was compared between the two groups. As there is a relationship between FGF21 and metabolic factors like cholesterol and triglycerides, these factors were also measured and analyzed. The data were analyzed with SPSS using the Chi-square and Mann–Whitney tests (α = 0.05). The two groups were matched for age and gender using the Kruskal–Wallis and Mann–Whitney tests, respectively. As the FGF21 data was not normally distributed, the nonparametric inconsistency test of Mann–Whitney was used. The required data were collected using a researcher-made questionnaire developed based on our research goals. This checklist included items on their history with pterygium, age group, gender, and serum level of FGF21. The research proposal was approved by the Research Council of the School of Medicine and the Committee of Ethics in Human Research at the Birjand University of Medical Sciences. The research goals and procedures were thoroughly discussed with each patient and assured they would remain anonymous and that their information would be kept confidential. Informed written consent was obtained from each patient. The participants could leave the study at any stage, with this not affecting their treatment.

##  RESULTS

This study consisted of 60 participants including 30 patients with pterygium and 30 healthy individuals forming the control group. No participants had any relevant medical history or were taking any medications that would impact our study's outcomes.

In the pterygium group, 96.7% of the patients had pterygium in the ocular nasal zone, 43.3% of patients had only one affected eye, while for the remaining 56.7%, both eyes were affected.

Patients with diabetes mellitus were excluded from the study. This was because it has been shown that the serum FGF21 level has a positive and negative correlation with type 2 and type 1 diabetes mellitus, respectively.

Regarding the comparison of serum FGF21 in patients with and without pterygium, the results of the Mann–Whitney test showed a statistically significant difference in the mean of the serum FGF21 between the pterygium (319.09 ± 246.93) and control (608.88 ± 449.81) groups (*P* = 0.005). Moreover, regarding the comparison of serum FGF21 in pterygium patients in terms of gender, the Mann–Whitney test indicated no significant difference between males (281.55 ± 40.74) and females (361.375 ± 10.298) (*P* = 0.198) [Table 2].

A regular diet one week before the sampling session was enforced to more accurately evaluate the lipid serum levels after a 12-h fasting period.

An independent *t*-test revealed a significant difference in the mean cholesterol levels between the cases (206.86 ± 50.77) and controls (155.53 ± 32.15), being significantly higher in patients with pterygium (*P* < 0.0001). The corresponding figures for LDL were 101.71 ± 30.62 and 76.00 ± 22.26 (*P* = 0.001). The results of the Mann–Whitney test further demonstrated a significant difference in triglyceride levels in patients with pterygium (138.66 ± 102.83) compared to the control group (98.90 ± 50.52; *P* = 0.03); however, there was no significant difference in terms of the HDL levels between the two groups (48.17 ± 22.0 and 57.33 ± 33.7, respectively;* P* = 0.2).

Liver and kidney functions were also assessed, as FGF21 secretes from and exerts through these two organs, respectively. The Mann–Whitney test suggested no significant difference in creatinine levels between the pterygium (0.92 ± 0.18) and control (0.95 ± 0.14) groups (*P* = 0.2). An independent *t*-test showed no significant difference in the urea level between the two groups (28.07 ± 7.94 and 24.43 ± 6.86, respectively;* P* = 0.07). The same test suggested no significant difference in the serum glutamic–oxaloacetic transaminase (SGOT) and serum glutamic–pyruvic transaminase (SGPT) levels between the case and control groups (*P* = 0.09 and *P* = 0.42, respectively) [Table 3].

##  DISCUSSION

Our findings showed a significant difference in serum FGF21 between the pterygium and control groups. The serum FGF21 was significantly lower in patients with pterygium compared to non-pterygium patients (*P* = 0.005). Fibrovascular proliferation, along with several inflammatory factors, are important in pterygium pathogenesis. Increased vascular endothelial growth factor (VEGF) levels have been reported in immunohistological studies, with bevacizumab being used to improve the outcomes and reduce recurrences of pterygium.^[12]^ However, FGF21 acts independently of VEGF.

Our findings regarding the effects of FGF21 on inhibiting angiogenesis are consistent with the results of the study by Fu *et al* in which they observed the inhibition of angiogenesis by systemically administering this factor to mice. This inhibitory effect was exerted through adiponectin and TNFα and was seen to be independent of VEGF.^[6]^ In humans, the administration of FGF21 increases adiponectin in a dose-dependent form.^[13]^ Low levels of adiponectin in human blood circulation may be associated with neovascularization-related diseases in the eyes.^[14,15]^ Our results are consistent with the protective effects of this factor in neovascularization-related eye diseases, as described by previous studies. Chavez *et al* compared the serum FGF21 in thin, fat, fasting blood sugar impaired and diabetic patients and found that people with type II diabetes had higher serum FGF21 compared to other groups, linking this to the status of insulin resistance.^[16]^ Also, in a study by Kralisch *et al*, the mean concentration of serum FGF21 in patients with type II diabetes mellitus was 2.1 times higher compared to the healthy controls.^[17]^ The level of this factor decreases in type I diabetes as it is synthesized in the liver and pancreas and people with type I diabetes have impaired pancreas function. The increased level of FGF21 in type II diabetes is due to the compensatory effect induced by insulin resistance in these patients.^[18]^

Therefore, despite the beneficial effects of this factor on lipid metabolism^[9]^ and reducing angiogenesis, it is asserted that tissue resistance to FGF21 in obesity^[19]^, fatty liver, and related metabolic disorders^[19,20]^ are responsible for an increased serum level of FGF21. The same pathogenesis is suggested regarding insulin function in type 2 diabetes mellitus.

This is similar to type 1 diabetes mellitus in which auto-immune degeneration of the pancreatic cells results in decreased insulin levels. In type 2 diabetes mellitus, the tissue resistance to insulin leads to hyperinsulinemia, and the physiologic level of insulin is not effective in controlling the serum glucose level.^[20]^

Given that the serum level of this factor is affected by various types of diabetes, people with diabetes were excluded from our study. Our findings further showed a significant difference in the mean cholesterol level, mean triglyceride level, and mean LDL level between the pterygium and control patients. The means of these three indices were significantly higher in patients with pterygium compared to the control group. This study took into account the probability of such metabolic changes in pterygium patients.

In line with our results, Malekifar *et al* ^[21]^ showed that pterygium is significantly related to ischemic heart disease and obesity. Accordingly, it can be suggested that pterygium is not an isolated disease and can present as a metabolic condition.^[22]^ However, further studies are needed to confirm this idea.

Other observations in South Korea revealed a relationship between obesity and pterygium. They showed that hyperlipidemia and obesity are more prevalent in patients with pterygium due to lower FGF21 serum levels.^[23]^ Moreover, consistent with our findings, a study by Wente *et al* showed that FGF21 reduced lipid levels and improved lipid metabolism in rats.^[24]^ In the study by Zhang *et al*, the administration of FGF21 to type I diabetics diminished apoptosis, improved lipid metabolism, reduced fat accumulation in the organs, and decreased oxidative stress. Overall, complications in these patients were reduced.^[8]^

In the current study, the reduced level of FGF21 in patients with pterygium was associated with impaired lipid metabolism. If future studies confirm this finding, pterygium can be considered a cardiovascular risk factor. The serum FGF21 level was not significantly different between males and females in our study, while in Zibar's study, the serum FGF21 was higher in type I diabetic females compared to males. However, the serum FGF21 was not different between males and females in the healthy subjects, a finding that is consistent with our results.^[25]^

We found no significant difference between the liver enzymes, urea, and creatinine between the two study groups. Since FGF21 is produced by the liver and excreted by the kidney, the difference of this factor between the two groups does not appear to be related to liver and kidney function.

In Panchapak's study conducted in Australia on 3,564 patients aged 49 years or older, 7.3% of the patients had pterygium and 69.5% had pinguecula, with the prevalence being significantly higher among males than females.^[26]^ The findings demonstrated that in more than 96% of the patients, the pterygium site was the ocular nasal zone. This is consistent with our study, in which pterygium was more likely to occur in the ocular nasal zone. Most patients had unilateral pterygium. Overall, 43.3% of patients were affected with pterygium in only one eye and 56.7% in both eyes. Finding appropriate pharmacological therapy options for patients who do not wish to undergo surgery is a significant priority.^[27]^ It is hoped that some non-surgical treatment methods and prophylactic procedures will soon be introduced.

Regarding the high prevalence of cardiovascular disorders and its resulting mortality and morbidity, there is growing attention toward new cardiovascular risk factors such as inflammatory cytokines and hair growth on the external ear, even in younger adults. The results of this study and its association with obesity and ischemic heart disease as seen in previous studies, suggest that pterygium should be considered a cardiovascular risk factor.^[28]^ If pterygium was included in cardiovascular screening tests, cardiovascular complications might be reduced.

In conclusion, our findings suggest that low serum FGF21 in patients with pterygium affects the condition and may be a protective factor. In addition, impaired lipid metabolism should be taken into account in these patients.

##  Financial Support and Sponsorship

Nil.

##  Conflicts of Interest

There are no conflicts of interest.
